# Intraperitoneal Bleeding After Ultrasound-Guided Transperineal Prostate Biopsy

**DOI:** 10.1155/2024/8819291

**Published:** 2024-11-06

**Authors:** Lisa Chapman, Sorena Keihani, Alejandro Sanchez

**Affiliations:** ^1^Division of Urology, Department of Surgery, University of Utah, HELIX Tower, 30 North Mario Capecchi Drive, Salt Lake City, Utah 84112, USA; ^2^Division of Urology, Department of Surgery, Huntsman Cancer Institute, Salt Lake City, Utah, USA

**Keywords:** hemorrhage, postoperative complications, prostate biopsy, prostatic neoplasm, transperineal approach

## Abstract

Transperineal prostate biopsy is becoming a popular approach in the diagnosis of prostate cancer. Urethral bleeding and urinary retention are the most common complications. We report a case of intraperitoneal bleeding after transperineal prostate biopsy in a patient with history of focal therapy for prostate cancer. The patient presented with dizziness, abdominal pain, and tenderness a few hours after the procedure. A computed tomography (CT) scan showed intraperitoneal bleeding. He was managed conservatively without needing any interventions or blood transfusion. Intraperitoneal bleeding is a possible, rare, and unexpected complication after transperineal biopsy especially in smaller prostates with prior procedures and scarring.

## 1. Introduction

Prostate biopsy is currently the standard of care for the initial diagnosis of prostate cancer. Both transrectal and transperineal approaches can be used to obtain ultrasound-guided biopsies, with or without MRI fusion [1]. Transperineal prostate biopsy has become more popular in recent years with presumed benefits of a lower risk of infectious complications as well as easier access to anterior lobe tissue [2]. Recent randomized controlled trials suggest comparable rates of complications for transrectal and transperineal approaches [3–5]. Prostate biopsy is generally a safe procedure. While pain, minor bleeding, and infection are the most common complications, more serious complications can occur [2, 6]. We report a case of intraperitoneal hemorrhage after a transperineal prostate biopsy.

## 2. Case Presentation

**Figure 1:**
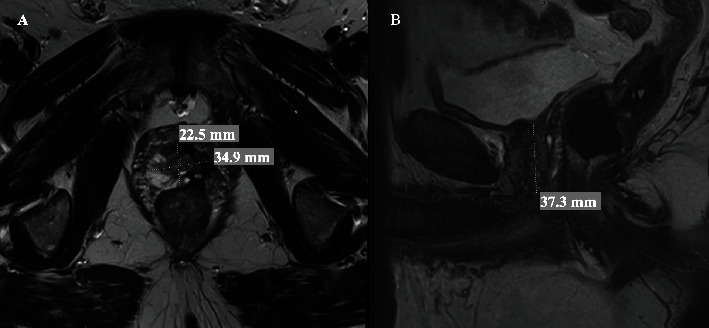
Axial (A) and sagittal (B) views of the multiparametric prostate MRI showing the prostate gland measurements with significant atrophy and total volume of 17 g.

The patient is a 66-year-old male with a history of gastroesophageal reflux disease and sleep apnea and a past surgical history of an open right inguinal hernia repair with mesh. Upon initial prostate cancer diagnosis in 2020, his prostate-specific antigen (PSA) was 10.8 ng/dL. A standard 12-core template transrectal prostate biopsy was obtained which was positive for Gleason 3+4 disease confined to the left base and mid prostate (4/12 cores positive, 60% and 70% percentages of tissue with carcinoma in each positive core, 10% total percentage of Pattern 4). His initial multiparametric MRI in 2020 showed a prostate size of 34 g with two 1.1 cm PIRADS (Prostate Imaging Reporting & Data System)-4 lesions in the left gland. He chose high-intensity focused ultrasound (HIFU) as focal therapy treatment for his prostate cancer in 2020. His nadir PSA after focal therapy was 1.2 ng/dL 1 year after the procedure. This gradually rose to 2.0, 2.6, and 3.2 ng/dL in the following 2 years. A posttreatment multiparametric prostate MRI was repeated in 2022 showing a 17-g prostate with evidence of atrophy and fibrosis in the left prostate, and without any suspicious lesions (PIRADS-2, Figure 1).

**Figure 2:**
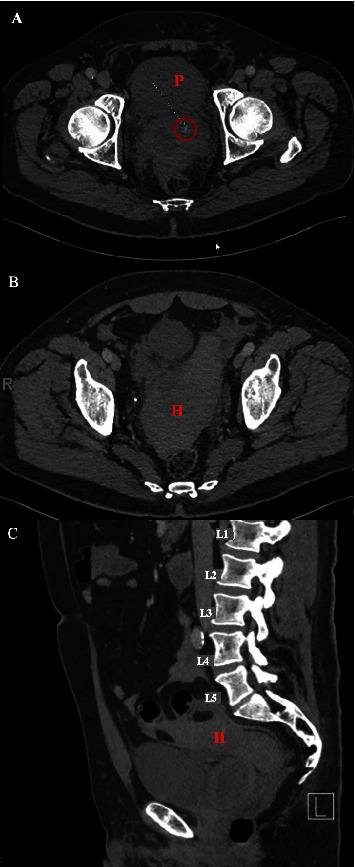
Intraperitoneal bleeding after transperineal prostate biopsy. (A) Axial imaging showing the prostate (letter P) and a small active contrast extravasation area (arrow and red circle). (B) Axial imaging showing the intraperitoneal hematoma (letter H). (C) Sagittal imaging showing the hematoma (letter H) above the bladder and tracking around the bowel.

Over the next year, his PSA rose to 4.5 ng/dL at which time a decision was made to proceed with a repeat biopsy to asses for residual/recurrent cancer. Given the lack of any targetable lesions in the previous MRI within the last year, a standard 12-core template transperineal biopsy was performed in the outpatient clinic setting and using local anesthetics without immediate complications, and then the patient was sent home. He returned to the emergency department about 2 hours after leaving the clinic due to significant dizziness and suprapubic and lower abdominal pain, which did not improve with a dose of Naproxen. He did not have any hematuria, rectal bleeding, or fevers. His initial vital signs were normal including a blood pressure of 133/66 mmHg and a heart rate of 66 bpm. His abdominal exam showed tenderness in the lower abdomen without distention or rebound. A complete blood count (CBC) was obtained showing a white blood cell (WBC) of 8.8 k/*μ*L and hemoglobin of 13.7 g/dL (from a baseline hemoglobin of 15.0 g/dL 6 months prior). Given his symptoms, a CT scan of the abdomen and pelvis with intravenous contrast was obtained. This showed a hematoma and fat stranding around the prostate with moderate volume of intraperitoneal hemorrhage. There was an area of arterial enhancement in the left prostate with pooling of contrast within the prostate on portal venous phase, suggestive of possible active contrast extravasation (Figure 2). Given that the patient was hemodynamically stable with a stable abdominal exam, he was admitted to the urology service for serial hemoglobin checks and abdominal exams. A 16-Fr. foley catheter was placed with 30 cc in the balloon, and this was placed on traction for an hour to possibly help with providing a tamponade effect for prostatic bleeding. His hemoglobin was checked every 6 h, which nadired at 9.1 after 48 h while his vital signs and abdominal exam remained stable. He was not tachycardic during his admission. His urine remained clear and the foley catheter was removed on admission Day 2. He was initially NPO while monitoring his symptoms, but then was allowed a regular diet starting the morning after presentation. He was ambulating at baseline and he was discharged from the hospital on Day 3 of admission with strict return precautions. He did not undergo any interventions or receive blood transfusions.

At clinic follow-up, 2 days after hospital discharge, he was recovering appropriately with significant improvement in his pain and abdominal exam. His hemoglobin was 11.0 ng/dL at this time. His prostate biopsy showed one core of Gleason 3+3 disease (1/12 cores positive, 10% of tissue with carcinoma) on the right base of the prostate, which suggested new metachronous lesion as the previous HIFU treatment was on the left side mid and apex of the prostate. Additionally, this would meet the criteria for acceptable treatment outcome in case of recurrence [7] and the patient opted for active surveillance. He was doing well on 2- and 6-month follow ups and his repeat PSA at 6 months remained stable.

## 3. Discussion

Prostate biopsy is a generally safe procedure with most of the complications being minor and self-limiting. The most common complications are bleeding (rectal/urethral), urinary retention, or infection [6]. Recent randomized trials comparing transperineal and transrectal prostate biopsy approaches have shown similarly low rates of complications and comparable rates of clinically significant prostate cancer detection [3–5]. The rates of emergency room visits and hospital admissions after prostate biopsy, regardless of approach, are well below 1% [4]. There are rare reports of significant rectal bleeding after a transrectal prostate biopsy which required blood transfusion and angioembolization or endoscopic clipping [8, 9]. However, hemoperitoneum (intraperitoneal bleeding) after transperineal prostate biopsy is a very rare and unexpected complication.

In our literature review, we found only one similar report of hemoperitoneum after a transperineal prostate biopsy [10]. Frascheri et al. [10] reported this bleeding complication after a transperineal biopsy in a 65-year-old man with a history of previous transurethral resection of the prostate (TURP). While their complication occurred after a 24-core biopsy, in contrast to our standard 12-core biopsy, there are some similarities between the two cases that warrant discussion. First, both patients had previous procedures (TURP in Frascheri et al.'s report and HIFU in this report) with the possibility of tissue scarring. Both patients had small prostates at the time of the biopsy (20 g vs. 17 g), which makes the biopsy procedure more challenging with the possibility of the needle extending beyond the prostatic tissue. Both patients were managed conservatively during their hospital admission although the patient in Frascheri et al.'s report required an ICU admission and received one unit of blood transfusion.

Frascheri et al. [10] postulated that puncture of the inferior vesical artery branches could occur with intraperitoneal tracking of the bleeding due to anatomical changes from previous procedures. We agree with this hypothesis and believe that the combination of scarring from the previous procedures (in our case HIFU), combined with a small-sized prostate, could lead to extraprostatic extension of the biopsy needle. Although an in-bore MRI-guided biopsy can in theory provide better visualization of the gland boundaries, we did not find any evidence that this would be beneficial for smaller glands or after focal therapy in the absence of a target lesion. The fibrosis/scarring makes it more difficult to insert the needle into the prostate itself and given the short length of the prostate after whole-gland treatments, there is a potential to go beyond the prostate/seminal vesicles with the base biopsies. The rectovesical pouch is the lowest anatomic point in the male peritoneal cavity. In the setting of previous procedures, the seminal vesicles and the Denonvillier's fascia could form adhesions to the peritoneum pulling this inferiorly and closer to the prostate. An injury to the branches of the inferior vesical artery (e.g., prostatic and seminal vesicle branches) can then lead to an intraperitoneal bleeding.

The vast majority of prostatic bleedings can be managed conservatively. These two reports suggest that conservative management can be used even in the case of intraperitoneal extension of the hematoma. If hemodynamic lability or instability results from active and continued bleeding, a CT angiography and interventional radiology consultation are warranted for possible angiography and angioembolization of the bleeding vessels. Exploratory laparotomy is reserved for significant bleeding with rapidly deteriorating hemodynamic instability.

## 4. Conclusions

Intraperitoneal bleeding is a very rare but potential complication with transperineal prostate biopsy in patients with prior prostate procedures or small glands. This can usually be managed conservatively although close monitoring and consideration of endovascular interventions are warranted.

## Data Availability

Additional data regarding this case is not publicly available in order to protect patient anonymity.

## References

[1] Wei J. T., Barocas D., Carlsson S. (2023). Early detection of prostate cancer: AUA/SUO guideline part II: considerations for a prostate biopsy. *The Journal of Urology*.

[2] Wilcox Vanden Berg R. N., George A. K., Kaye D. R. (2023). Should transperineal prostate biopsy be the standard of care?. *Current Urology Reports*.

[3] Hu J. C., Assel M., Allaf M. E. (2024). Transperineal versus transrectal magnetic resonance imaging-targeted and systematic prostate biopsy to prevent infectious complications: the PREVENT randomized trial. *European Urology*.

[4] Mian B. M., Feustel P. J., Aziz A., Kaufman R. P., Bernstein A., Fisher H. A. G. (2024). Clinically significant prostate cancer detection following transrectal and transperineal biopsy: results of the prostate biopsy efficacy and complications randomized clinical trial. *The Journal of Urology*.

[5] Gilberto G. M., Arcuri M. F., Falsarella P. M., Mariotti G. C., Lemos P. L. A. N., Garcia R. G. (2023). Complication rates of transrectal and transperineal prostate fusion biopsies - is there a learning curve even in high volume interventional center?. *International Brazilian Journal of Urology*.

[6] Borghesi M., Ahmed H., Nam R. (2017). Complications after systematic, random, and image-guided prostate biopsy. *European Urology*.

[7] Tay K. J., Amin M. B., Ghai S. (2019). Surveillance after prostate focal therapy. *World Journal of Urology*.

[8] Ando T., Watanabe K., Mizusawa T., Katagiri A. (2018). Late-onset rectal bleeding with hemorrhagic shock after transrectal prostate needle biopsy. *Urology Case Reports*.

[9] Dave N., Esmail Khan Ghasri R., Gonzalez H. H., Kaplan S. (2023). Hemorrhagic shock after transrectal ultrasound-guided prostate biopsy successfully treated with endoscopic therapy. *ACG Case Reports Journal*.

[10] Frascheri M. F., Contreras P., Blas L., Bonanno N., Ameri C. (2022). Hemoperitoneum after transperineal prostate biopsy. *Medicina (Buenos Aires)*.

